# Curvature geometry in 2D materials

**DOI:** 10.1093/nsr/nwad145

**Published:** 2023-05-18

**Authors:** Nan Wei, Yiran Ding, Jiaqian Zhang, Linyi Li, Mengqi Zeng, Lei Fu

**Affiliations:** College of Chemistry and Molecular Sciences, Wuhan University, Wuhan 430072, China; The Institute for Advanced Studies, Wuhan University, Wuhan 430072, China; College of Chemistry and Molecular Sciences, Wuhan University, Wuhan 430072, China; College of Chemistry and Molecular Sciences, Wuhan University, Wuhan 430072, China; College of Chemistry and Molecular Sciences, Wuhan University, Wuhan 430072, China; College of Chemistry and Molecular Sciences, Wuhan University, Wuhan 430072, China; The Institute for Advanced Studies, Wuhan University, Wuhan 430072, China

**Keywords:** 2D materials, curvature geometry, deformation, strain

## Abstract

The two-dimensional (2D) material family can be regarded as the extreme externalization form of the matter in the planar 2D space. These atomically thin materials have abundant curvature structures, which will significantly affect their atomic configurations and physicochemical properties. Curvature engineering offers a new tuning freedom beyond the thoroughly studied layer number, grain boundaries, stacking order, etc. The precise control of the curvature geometry in 2D materials can redefine this material family. Special attention will be given to this emerging field and highlight possible future directions. With the step-by-step achievement in understanding the curvature engineering effect in 2D materials and establishing reliable delicate curvature controlling strategies, a brand-new era of 2D materials research could be developed.

## Teaser

Curvature engineering offers a new tuning freedom beyond the thoroughly studied layer number, grain boundaries, stack, etc. The precise control of the curvature geometry in 2D materials can redefine this material family.

## INTRODUCTION

**Figure 1.**
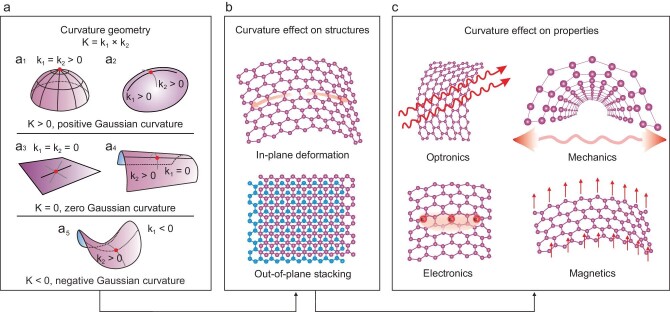
Curved geometry and property variation in 2D materials. (a) Three types of Gaussian curvature. (b and c) the influence of curvature on structures and properties of 2D materials.

The two-dimensional (2D) material family can be regarded as the extreme externalization form of matter in the planar 2D space. Proving this is capable of excluding a variety of types of ordering in one and two dimensions. Almost perfect crystals must exist in three dimension (3D) systems. In other words, 2D materials can only exist in a stable fashion if they crimp in 3D space, so as to suppress thermal vibration and minimize total free energy. Benefiting from the atomically thin structure and high mechanical strength superiority, these materials can withstand intensive external stress. The structure that remains intact under external strain allows us to adjust its curved geometry on a larger scale. Curvature technology offers a new tuning freedom beyond the thoroughly studied layer number, grain boundaries, stacking order, etc. There is no doubt that precise control of the curvature geometry in 2D materials can redefine this material family. Generally, the Gaussian curvature of a point on the surface is the product of its principal curvatures k_1_ and k_2_ (K = k_1_ × k_2_). By this definition, there are three types of curvature geometry in 2D materials: positive Gaussian curvature (K > 0), zero Gaussian curvature (K = 0) and negative Gaussian curvature (K < 0) (Fig. 1a). Curvature geometry is a kind of topological structure. The atomically thin property and weak van der Waals (vdW) interaction of 2D materials allow in-plane deformation and out-of-plane stacking (Fig. 1b). Such a geometry deformation will significantly lead to strain introduction, symmetry variation and defect formation of 2D materials, resulting in the strong perturbation of band structure, interlayer coupling and phonon vibration, which will give new expression to their electrical, optical, magnetic and mechanical properties [1–5] (Fig. 1c). Precisely engineering the curvature geometry in 2D materials can undoubtedly bring in a new era for this technology.

Curvature is the intrinsic structure of 2D materials before 2D materials actually became 2D materials. So far, relevant studies about curvature in 2D materials mainly focus on the fundamental theoretical study or experimental realization using accurate equipment. Achieving the synthesis of 2D materials with precise curvature control is still a challenge. More often than not, planar substrates were adopted for crystal synthesis to achieve lattice-matched and layer-by-layer growth. Programming the growth of 2D materials with the designed curved structure demands delicate curvature engineering of the growth interface. Recently, some pioneers have taken a step forward, involving the curvature-stabilized ABC three-layer graphene (TLG) growth [6], super-twisted spirals of layered materials [7] and curvature engineered bandgap tuning of 2D materials [8]. Curvature geometry in 2D materials is not a new concept, but it is a brand-new starting point for the development of 2D materials. Research of curvature geometry in 2D materials is in its infancy, and there have been no reliable delicate curvature controlling strategies for large-scale application so far. This perspective is not only a discussion on the regression of 2D materials in terms of their curved nature but also a new view of its development direction. Nevertheless, this topic has, so far, received little attention. A timely revisiting and well-thought-out perspective could potentially fill such a gap. The precise synthesis of 2D materials in curved geometry will set off a brand-new wave of interest in the near future, which might be another classical case as in the chirality to carbon nanotubes.

In this perspective, first, we focus on the regulation of 2D material curvature geometry, including ripple and fold, buckle and pucker, defect, deformation, etc. Subsequently, we will introduce the influence of curvature on 2D material structure, mainly encompassing in-plane deformation and out-of-plane stacking. Additionally, the curvature effect on properties of 2D materials will be discussed in detail, including optical, electrical, mechanical and magnetic properties. Finally, we intend to display insight into the field of curvature geometry in 2D materials from four aspects, covering the designability of curvature, construction of new materials, stability of curvature, as well as the study of the mechanism of the curvature effect. With the step-by-step achievement in understanding the mechanism of the curvature engineering effect in 2D materials and the establishment of reliable precise curvature controlling strategies, a brand-new era of 2D materials research could be developed.

## CURVATURE GEOMETRY REGULATION

In earlier studies of graphene, intrinsic elastic ripples on its surface stabilized this high-quality 2D crystal and were associated with the high mobility of charge carriers within it. Such encouraging achievements indicate that curvature regulation is an effective method of tuning the structure of 2D materials. In this section, we focus on the regulation of 2D material curvature geometry, including ripple and fold, buckle and pucker, defect, deformation, etc.

### Ripple and fold

**Figure 2.**
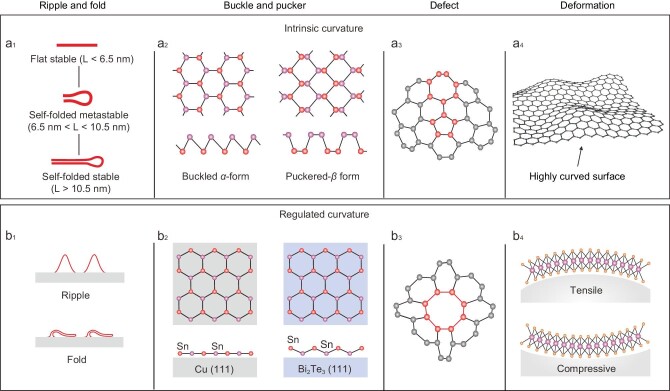
Curvature geometry regulation in 2D materials. (a) Intrinsic curvature in 2D materials. (b) Regulated curvature geometry in 2D materials.

When the length or width of 2D material exceeds the critical value (10.5 nm for graphene), it spontaneously forms a fold structure due to thermal instability (Fig. 2a_1_) [9]. Researchers are now working on how to manipulate these ripples and folds in a controlled way by controlling growth temperature and selecting appropriate substrates. Due to the different thermal expansion coefficients between material and substrate, the strain will accumulate in the growth material plane during the process of rising and cooling, resulting in ripples and folds (Fig. 2b_1_) [10]. These textures can also be manipulated to bifurcate and fuse through annealing treatment. The introduction of ripples and folds in other 2D materials can also reduce electron-phonon scattering and thus improve their carrier mobility, which can be used in the manufacture of high-performance room-temperature field-effect transistors and thermoelectric devices.

### Buckle and pucker

Periodic ripple can be achieved when appropriate post-processing is chosen [11]. However, ripples and folds are usually regarded as local deformations, which are often random and uncontrollable. Completely uniform curvature geometry regulation across the whole 2D material can fundamentally change its structural properties. This is particularly common in heavier element systems. For example, compared with graphene, silicon in the same group is unable to form robust π bonds due to its longer bond lengths compared with graphene (2.28 Å vs 1.42 Å). In order to overlap in its p_z_ orbitals (responsible for the π bond), silicene forms a buckled structure that stabilizes its honeycomb construction. Similarly, the puckered structure is also an intrinsic geometric configuration for 2D materials (Fig. 2a_2_) [12]. This special geometric curvature enhances the spin-orbit coupling of the materials, thus opening the bandgap and allowing them to exhibit the quantum spin Hall effect. The adjustment of these special geometries is global. Therefore, a perfectly uniform force is necessary. Bonding or electron transfer between the substrate and the material can also force changes in the original atomic spatial distribution [13], as seen in Fig. 2b_2_.

### Defect

Defects will cause the loss of atom coordination, resulting in rearrangements and fluctuations of atoms, which locally appear as a curved structure. Defects can be introduced into 2D materials by means of external stress, surface adsorption and doping. As seen in Fig. 2a_3_, at the intrinsic five-seven ring of graphene, the curvature structure automatically forms as the atom leaves its most stable position.

Furthermore, the precise control of nanoscale curvature can be achieved through asymmetric structure design approaches. For example, through the Diels–Alder reaction and subsequent Scholl reaction in the organic synthesis process, the circulene can be locally embedded in the graphite lattice (Fig. 2b_3_). Negative curvature of 3D twisted nanographene structures can be obtained via distortion strain introduction [14]. The curved interface generally leads to the synthesis of 2D curved materials with micro-scale curvature changes. For higher-resolution curvature construction in the 2D material, designing the asymmetric structure can induce curved structures at the molecular and even atomic levels.

### Deformation

Two-dimensional materials, especially nonlayered materials with high surface energy, exhibit intrinsic curvature, even to the extent of micrometer scale (Fig. 2a_4_). All the curvature regulation methods mentioned above are irreversible. An external field tuning provides a facile and reversible route to introduce curvature geometry in 2D materials. Homogeneous and integral deformation can be programmed by a well-designed interface, for example, a curved or flexible substrate. By means of adjusting the diameter and positive/negative curvature of the self-reshaping curved substrate, the bandgap can be adjusted linearly with the diameter of substrate curvature (Fig. 2b_4_) [8]. Using the tip of a needle to apply an external field in order to induce curvature deformation of 2D materials is another typical method [15]. The suspended 2D material will undergo severe deformation under the force of the needle tip, the degree of deformation is determined by the external force. One of the advantages of this method is that the influence of curvature on the properties of 2D materials can be qualitatively analyzed. Recently, this local deformation strategy has been applied to WSe_2_. Because the dark exciton in the WSe_2_ is affected by out-of-plane deformation, the peak of the dark exciton is redshifted by more than 390 meV in the PL spectrum when the applied stress reaches 5% [16].

Besides these mechanical stimulations, applying an electrical or thermal field are also common strategies, especially for weak vdW coupling and anisotropy materials [17,18]. When exposed to these external fields, 2D materials exhibit complex curvature variation behaviors, such as bending, curling, periodic wrinkles, and so on.

Therefore, profiting from these results, curvature geometry provides a promising route to fabricate complex and atomically precise curvature nanostructures in 2D materials. However, research of curvature geometry in 2D materials is still in its infancy. Developing reliable curvature-controlling strategies for large-scale applications is of great importance and highly desirable. This will allow us to build curvature engineering on demand rather than empirical optimization.

## CURVATURE EFFECT ON STRUCTURES

**Figure 3.**
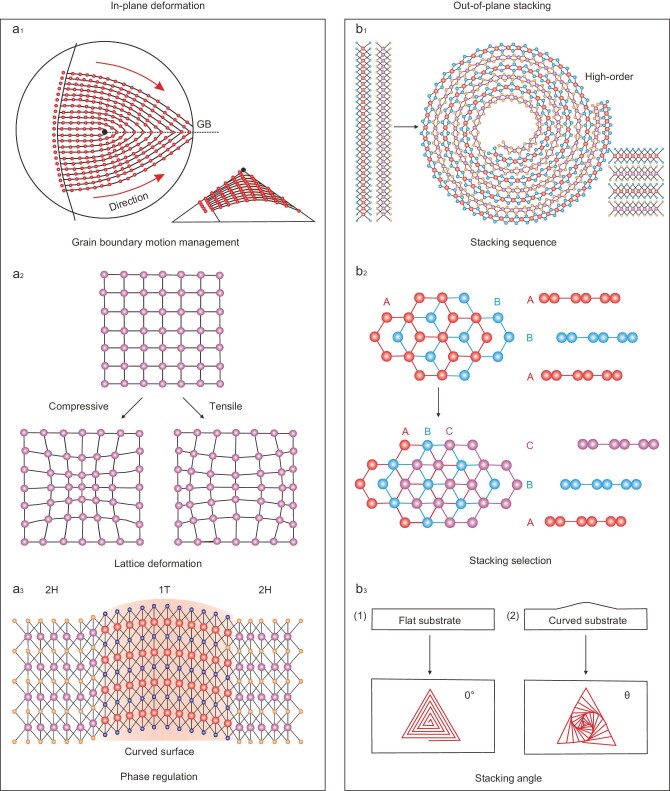
Curvature effect on the structure of 2D materials. (a) Effects of curvature-induced in-plane deformation, including grain boundary motion management, lattice deformation, and the phase transition. (b) Effects of curvature-induced out-of-plane stacking, including stacking sequence, stacking selection and stacking angle.

Atomically thin characteristics enable 2D materials to have excellent designability and can exhibit abundant curvature structure, which will significantly affect the atomic configurations. To understand the effects of curvature on the structure of 2D materials, we review the influence of curvature on 2D material structures from two aspects, including in-plane deformation and out-of-plane stacking. Meanwhile, the progress toward controlling growth behavior will be systematically elaborated. The influence of in-plane deformation covers grain boundary motion management, lattice deformation, and phase transition (Fig. 3a). The out-of-plane stacking structure will influence the stacking sequence, stacking sequence selection and stacking angle (Fig. 3b).

### In-plane deformation

One of the curvature-induced in-plane deformations is the change of motion mode of the grain boundary. Recently, theoretical work has predicted that with the aid of delicate regulation of curvature geometry, the position, finite length and misfit angle of grain boundary can be highly controlled with favorable designability (Fig. 3a_1_) [19]. Grain boundary management engineered into 2D materials could potentially introduce desired properties, such as magnetism and enhanced light response. To our knowledge, the atomically thin property of 2D materials ensures a uniform covering on the positive/negative curvature substrate, resulting in lattice deformation. For instance, curvature introduction will trigger lattice tension/compression deformation (Fig. 3a_2_), thus leading to the adjustment of its bandgap [20]. This is of great significance to the application of 2D material-based devices. The curvature effect also involves phase transformation. Recently, a variety of binary compounds have been prepared by surface alloying strategy on gold-based alloys. During the process of selective etching of nanoporous gold, unsaturated coordination and structural distortion can be induced by a high curvature gradient. Significantly, since the change in the coordination configuration of atoms, part of the 2H phase will change into 1T phase and form an in-plane 1T-2H heterostructure, which is beneficial to multiphase electrocatalysis (Fig. 3a_3_) [21].

### Out-of-plane stacking

Stacking 2D materials with different properties together to form a vdW heterostructure can regulate physical properties at the atomic level [22]. By controlling the twist angle between the atomic layers, some fascinating quantum phenomena will be generated. Curvature engineering provides a reliable route for achieving precise and efficient stacking regulation by tailoring the substrate curvature levels and geometry configuration. Recent work has reported a novel approach to fabricating high-order vdW superlattices by means of curvature engineering. When vdW heterostructures were exposed to ethanol-water-ammonia solutions, the spontaneous delamination and rolling-up process can be driven by capillary force, resulting in diverse high-order vdW superlattices (Fig. 3b_1_) [23]. These superlattice structures can change the electronic band structure and realize the modulation of stacking sequence, which is of great significance to the fundamental research and industrial application of vdW heterostructures. In general, 2D materials have a stable stacking configuration in their natural state. Curvature engineering can stabilize some metastable configurations. For example, three layers of graphene are stacked in semi-metallic ABA or semiconductor ABC configurations, where the ABC configuration is metastable. Nevertheless, the unstable ABC configuration can be stabilized by a substrate with corrugated curvature because the curvature of the substrate will cause in-plane interlayer strain, and the subsequent layer must be stretched (compressed) to accommodate this change (Fig. 3b_2_) [6]. Curvature engineering provides a promising method for stacking sequence selection of 2D materials. The fabrication of graphene with a specific stacking angle is extremely demanding if its quantum properties want to be used in practical devices. The curvature can induce certain twists between layers exploiting substrate curvature design. A striking example is the growth of spiral dislocated layered materials on non-Euclidean surfaces. The weak vdW interaction allows relative movement between layers. Moreover, there is a geometric mismatch between the non-Euclidean surface and the Euclidean lattice, where the Euclidean lattice bends with the non-Euclidean surface, with each distortion producing additional lattice distortion. Simultaneously, spiral dislocations constantly generate new interface layers and ensure the twist angle between each layer is consistent. As a result, fascinating structures and properties can be produced by constructing different stacked structures and designing the morphology of the interface (Fig. 3b_3_) [7]. In addition, recent work shows that some unique new optical properties will emerge from continuously-twisted 2D materials grown on curved surfaces due to C_3_ symmetry breaking caused by the strongly asymmetric charge density differences in the moiré supercell arising from a nontrivial interlayer coupling [24]. This novel design of substrate curvature can facilitate the discovery of more new physical properties in the near future. More generally, helical structures can be obtained under low saturation growth conditions, which has been demonstrated for graphene, TMDs, LDH and other materials [25].

Curvature geometry introduces new degrees of freedom into the traditional 2D material growth system, creating opportunities for the construction of new structures in a designed fashion. Inspired by these significant advances, great effort should be devoted to offering a thorough understanding of the curvature-induced structural response, aiming to provide direct guidelines for structural optimization design of the curvature engineering of 2D materials.

## CURVATURE EFFECT ON PROPERTIES OF 2D MATERIALS

The properties of 2D materials exhibit high responsiveness to structural variation, and the curvature geometry can strongly influence the lattice and electronic structure, thus the electrical, optical, magnetic and mechanical properties can be controlled effectively by curvature engineering. These curvature-induced changes can allow us to create materials with vastly different properties at the nanoscale and offer access to improve the performance of 2D materials-based devices, including photonic devices, solar cells, strain sensors, stretchable electrodes, flexible field-effect transistors, wearable devices, spintronic devices and so on.

### Optical property

**Figure 4.**
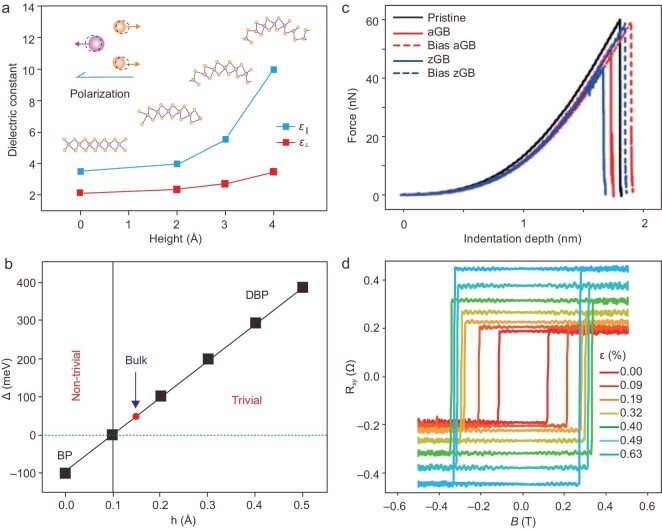
Curvature effect on the property of 2D materials. (a) Normalized photoluminescence spectra of black phosphorus under zero strain, compressive strain and tensile strain. Adapted with permission from ref. [28]. Copyright 2022, Springer Nature. (b) Current-voltage curves of the buckling Te-based devices under different strains in [0001] direction. Adapted with permission from ref. [29]. Copyright 2015, American Chemical Society. (c) Relationship between indentation force depth and grain boundary of graphene film. Adapted with permission from ref. [30]. Copyright 2015, American Chemical Society. (d) Strain-induced changes in the hysteresis loop. Adapted with permission from ref. [31]. Copyright 2020, Wiley.

Introducing curvature into 2D materials enables bandgap modulation, which directly determines the optical properties of 2D materials such as light absorption and emission. By regulating the curvature to change the spatial distribution of strain, the efficient [8,26], precise local [27] modulation of bandgap can be achieved. Owing to the strong coupling of curvature with various internal degrees of freedom, including charge, photon and spin, this ability opens up novel possibilities for exploring interesting physics in 2D limits. With bending of the 2D material, the dielectric constant changes and inhibits phonon scattering, which greatly increases carrier mobility (Fig. 4a) [28].

### Electrical property

The curvature of 2D materials can induce structural deformation. As the properties exhibit high responsiveness to the structural variation, the carrier mobility of curved 2D materials, in the presence of strain, can be changed. An increase of two orders of magnitude in room temperature mobility was observed in folded MoS_2_. Besides the electronic property, band structure is also closely bound up to curvature. Due to the planar structure of graphene, the first-order spin orbital coupling is turned off, resulting in a negligible bandgap. However, when the bulked structure is introduced into the 2D materials, the π and σ orbitals of the nearest neighbor atoms will be hybridized, thus opening the bandgap (Fig. 4b) [29].

### Mechanical property

Curvature can bring rich mechanical properties to 2D materials. Notably, the curvature can induce the formation of topological defects (such as grain boundaries) and trigger different mechanical responses of 2D materials under different types of curvature, resulting in admirable mechanical properties. For instance, theoretical work has predicted that in a polycrystalline graphene system, the local mechanical response can be stiffened and strengthened by topological defects under a positive curvature, while softened and weakened mechanical responses are shown at locations with negative curvatures (Fig. 4c) [30]. The curvature geometry of topological defects adds a new degree of freedom to the design and modification of 2D materials.

### Magnetic property

Compared with traditional magnets, the physical parameters such as anisotropy energy and spin exchange of 2D magnets are sensitive to lattice deformation. In 2D magnets, coercivity, Curie temperature and even magnetization inversion (such as paramagnetism to ferromagnetism) can be controlled by effective adjustment of the easily magnetized axis with the aid of curvature geometry. Figure. 4d shows the Hall resistance versus the external magnetic field under different tensile strains. The change of in-plane deformation can lead to the enhancement of spin coupling, resulting in the gradual increase of coercivity [31]. Therefore, curvature engineering plays a significant role in extending the functions of spintronic devices, which can induce a series of fascinating phenomena such as ultrasensitive magnetization reversal [31–33].

## CONCLUSION AND PERSPECTIVE

With the aid of precise regulation of curvature geometry, 2D materials exhibit emerging structures and excellent properties. Although some significant progress has been achieved in the curvature-controlled 2D material system, there still exist many challenges and opportunities, including the designability of curvature, construction of new materials, stability of curvature, as well as the study of the mechanism of the curvature effect.

### Designability of curvature

**Figure 5.**
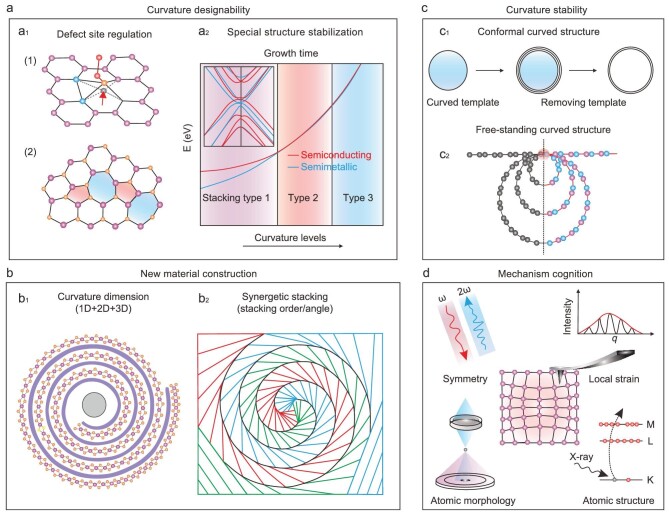
The potential prospect of curvature engineering. (a) Fine curvature regulation. (b) New material construction. (c) Curvature stability. (d) Mechanism of the curvature effect.

The curved substrate can directly influence the structure, physicochemical properties and growth behavior of 2D materials. More abundant and designed curved structures in 2D materials can be expected, such as periodic or 3D distributed curved structures. Taking a three-dimensional distributed curved carbon structure as an example, the geometric bending of the carbon skeleton will induce local strain, which is negatively correlated with the curvature of the carbon skeleton, making it easy to adjust the local strain (Fig. 5a). Carbon allotropes and graphene-related 2D materials arguably have the richest curved structures due to the more flexible C sp^2^ or sp^3^ bonding. At the atomic level, sp^2^- and sp^3^-bonded C atoms are associated with line defects and screw dislocations that resolve topological complexities. This provides insight into the synthesis of various 2D curved carbon materials [34]. In addition, it has been found that when the single-atom catalyst was coupled with this curved carbon skeleton, the rational design and construction of curvature will contribute to the structural distortion in the single-atom site, facilitate the electron transfer of the active site, and significantly improve the activity of the catalytic reaction (Fig. 5a_1_) [35]. In curvature engineering, researchers usually avoid inelastic strain relaxation due to dislocation plasticity or fracture. However, defects are important for catalysis. Using stress-assisted defect nucleation, catalytic active sites, such as the activation of inert MoS_2_ in-plane sites, can be artificially created for HER reactions. Defect sites in 2D materials generating on-demand under the guidance of the geometry of growth substrates, for example, can produce periodic defects that may have synergistic effects which can make great sense in the field of catalytics.

It is found that substrate curvature contributes to the formation of interlayer dislocations. At the same time, the curvature can stabilize some interlayer dislocations. Certain types of stable dislocations imply the presence of metastable configurations (such as the metastable semiconducting ABC configuration mentioned above). When the substrate curvature is adjusted to an appropriate range, the desired stacking configuration can be regulated on demand (Fig. 5a_2_) [6]. This flexible substrate curvature designability can facilitate the triggering of new growth behavior of 2D materials and the digging of novel properties.

### Construction of new materials

Curvature engineering can broaden the 2D material library. Novel curved 2D materials that would result from poking, bending or folding like a piece of paper can be fully expected. These geometry structures will lead to property evolution or even brand-new properties, offering access to redefine the 2D material, i.e. a new 2D material family. In addition to conventional 2D materials, curvature geometry can be combined with other low-dimensional materials to create mixed-dimensional vdW heterostructures with an infinite number of combinations. It has been reported that many quantum phenomena, including unconventional superconductivity and moiré excitons, are observed by adjusting the twist angle between stacked 2D materials. However, this relies heavily on labor-intensive physical stacking methods and the stacking angle of the product is extremely uneven. There is no doubt that heterogeneous structures with different stacking angles can be easily obtained by the nano-coil strategy, and even periodic superlattice structures composed of a variety of materials can be created. At present, the creation of 2D superlattices is still faced with problems such as alloying and inhomogeneity. It is expected that many new physical phenomena will be discovered by curvature-induced mixed-dimensional heterostructures with the designing stacking sequence and interlayer twist angle [7,23] (Fig. 5b).

### Stability of curvature

The dimension of the curved structure can range from several atoms to micro scales, which will result in different quantum effects in 2D materials. Besides, the stability of the obtained curved 2D materials after releasing the applied external field or removing the conformal growth substrate also deserves our attention. The scale and precision of the curvature can be important for the stability of the 2D curved structure. It has been reported that graphene shells grown on the surface of curved nickel metal nanoparticles can well remain in the curved hollow spherical structure with great stability (Fig. 5c_1_) [36]. It follows strong interactions between substrate and material, including bonding and charge transfer, which are fundamental to ensure material curvature engineering, thus introducing sufficiently large stresses. Therefore, the curvature of the material can be designed in terms of both alloying effect and element electronegativity difference. For example, there is a strong bonding tendency between S and Au. Au will not wet carbon materials, so when reaching its melting point, Au will spontaneously form a sphere in these carbon substrates. When TMDs grow on their surface, TMDs will be strongly deformed. This strategy can theoretically be extended to all 2D material systems. Considering the electronegativity difference between the substrate and the material, the electron transfer process can be understood, and the electron cloud density and bonding mode of the material can be controlled to obtain different buckled structures. For more general systems, such as graphene on flexible substrates, it is difficult to introduce more than 1% biaxial stress into them. Therefore, the way to introduce curvature should be more concentrated, for example, by using a tapered substrate. In theory, graphene can withstand up to 25% biaxial stress, provided there are no defects. And, as for the single layer or non-layered 2D materials, the curvature of the substrate can be completely duplicated onto the material, thus introducing sufficient strain.

In addition, through first-principles calculations, a series of G-BN heterostructures with atomically constructed curved structures can be designed (Fig. 5c_2_) [37]. Therefore, the curvature engineering of heterojunction without slip is also the focus of future research, which is important in the field of optoelectronics. Maintaining the curvature effect in 2D materials and improving the durability of curvature in devices are significant to meet different application requirements.

### Study of mechanism

*In situ* characterization can provide an in-depth study between material curvature and properties, gaining fundamental mechanism insights (Fig. 5d). The curvature will induce spatially heterogeneous strains, which need to be studied using local probes. The atomically high-resolution microscope can be used to characterize the microstrain effect and identify the presence of curved lattice structures and defects. Synchrotron radiation techniques can also be used to characterize material structure and property in detail, such as coordination structures and electronic states. The second harmonic generation signal is highly sensitive to the structural symmetry changes of 2D materials and can be used to characterize the limit of strain tolerance induced by curvature geometry. Especially, the extremely high curvature and the possible resulting anisotropy effect should be focused on. For instance, on the curved substrate, within the tolerance range, a single crystal can accommodate considerable local strain. When the curvature reaches a certain range, grain boundary or fractured growth in different directions can be induced [38]. Therefore, it is very important to design substrates with different curvature levels and explore the mechanism of the curvature effect combined with advanced characterization techniques, involving the measurement of local strain, observation of atomic morphology/structure and change of structural symmetry, etc. In summary, curvature geometry provides a new platform for exploring new growth behaviors and mechanisms of 2D materials.
